# The cross-tissue metabolic response of abalone (*Haliotis midae*) to functional hypoxia

**DOI:** 10.1242/bio.031070

**Published:** 2018-03-15

**Authors:** Leonie Venter, Du Toit Loots, Lodewyk J. Mienie, Peet J. Jansen van Rensburg, Shayne Mason, Andre Vosloo, Jeremie Z. Lindeque

**Affiliations:** 1 Human Metabolomics, North-West University, Potchefstroom Campus, Private Bag X6001, Potchefstroom 2520, South Africa; 2 School of Life Sciences, University of KwaZulu-Natal, Westville Campus, Private Bag X54001, Durban 4000, South Africa

**Keywords:** Abalone, Functional hypoxia, Metabolism, Metabolomics

## Abstract

Functional hypoxia is a stress condition caused by the abalone itself as a result of increased muscle activity, which generally necessitates the employment of anaerobic metabolism if the activity is sustained for prolonged periods. With that being said, abalone are highly reliant on anaerobic metabolism to provide partial compensation for energy production during oxygen-deprived episodes. However, current knowledge on the holistic metabolic response for energy metabolism during functional hypoxia, and the contribution of different metabolic pathways and various abalone tissues towards the overall accumulation of anaerobic end-products in abalone are scarce. Metabolomics analysis of adductor muscle, foot muscle, left gill, right gill, haemolymph and epipodial tissue samples indicated that South African abalone (*Haliotis midae)* subjected to functional hypoxia utilises predominantly anaerobic metabolism, and depends on all of the main metabolite classes (proteins, carbohydrates and lipids) for energy supply. Functional hypoxia caused increased levels of anaerobic end-products: lactate, alanopine, tauropine, succinate and alanine. Also, elevation in arginine levels was detected, confirming that abalone use phosphoarginine to generate energy during functional hypoxia. Different tissues showed varied metabolic responses to hypoxia, with functional hypoxia showing excessive changes in the adductor muscle and gills. From this metabolomics investigation, it becomes evident that abalone are metabolically able to produce sufficient amounts of energy when functional hypoxia is experienced. Also, tissue interplay enables the adjustment of *H. midae* energy requirements as their metabolism shifts from aerobic to anaerobic respiration during functional hypoxia.

This article has an associated First Person interview with the first author of the paper.

## Abstract (precis)

**Summary:** We report, for the first time, a metabolic map of abalone metabolism in response to functional hypoxia, compiled from results obtained by metabolomics analysis.

## INTRODUCTION

Muscle performance is essential to the lifestyle of animals (Pörtner, 2002). Typically, intense bouts of muscle activity deplete cellular energetic reserves, resulting in increased oxygen demand beyond the rate of uptake (Morash and Alter, 2016), which limits adenosine triphosphate (ATP) production via muscle oxidative metabolism (Storey, 2005). During such burst contractile muscle activity, organisms are generally fuelled by anaerobic metabolism where ATP production is made possible from substrate-level phosphorylation via the breakdown of phosphagens, but also due to glycolytic degradation of, predominantly, carbohydrates (Baldwin and England, 1982; Ellington, 1983). While lipids and proteins are more ideally used for structural elements of cells, they can also assist with ATP supply to working tissues (Venter et al., 2016b). The ability of organisms to use alternative metabolic ways to meet metabolic demand when aerobic respiration is compromised, ensure survival until recovery from the anaerobic episode is possible, whereafter normal aerobic respiration can continue (Fields, 1983).

Abalone, single-shelled marine molluscs, are highly dependent on anaerobic metabolism during episodes of unfavourable oxidation (Morash and Alter, 2016), like functional hypoxia. Functional hypoxia occurs when the internal oxygen pressure in an organism falls due to intensive muscular activity (Liu et al., 2014). In natural environments this type of muscle activity is likely to occur when animals are trying to escape from predators, or are in the pursuit of prey. In such scenarios, the rate of ATP use by muscles is too high to be met by aerobic respiration, forcing the organism to rapidly switch to anaerobic respiration for ATP production in contracting muscles (Müller et al., 2012). In a typical abalone farming environment, extreme functional hypoxia scenarios are less common but can be induced when abalone climb and crawl for feeding purposes; or as a result of shell adhesion or righting after being dislodged (Morash and Alter, 2016). Increased single end-point metabolites of functional hypoxia in abalone are well known and attest to the fact that abalone breakdown phosphagens (like phosphoarginine) and utilise anaerobic glycolysis for energy production (Gäde and Grieshaber, 1986; O'omolo et al., 2003). However, current knowledge on the holistic metabolic response for energy metabolism during functional hypoxia, and the contribution of different metabolic pathways towards the overall accumulation of anaerobic end-products in abalone are far from clear. Furthermore, the organismal response associated with functional hypoxia has not been investigated in different abalone tissues, allowing for more uncertainty of how various tissues work together to ensure energy production and/or survival of abalone.

To this end, knowledge of the basic biochemical constituents of abalone under culture conditions would be a very useful tool in their management in aquaculture systems (Laas and Vosloo, 2010). Considering that *Haliotis midae* is an important aquaculture species in South Africa and the largest generator of revenue for the mariculture sector (Dale-Kuys et al., 2017), insights into the metabolic alterations to external stressors are crucial for optimising farming strategies. With this being said, metabolomics research in aquaculture is proving extremely valuable for generating novel information into the mechanisms implemented by aquatic organisms (Young et al., 2017). Metabolomics can be defined as the nonbiased identification and quantification of metabolites in a biological system, using highly selective and sensitive analytical techniques (Dunn et al., 2005). Metabolites, or small molecules within a cell, tissue, organ, biological fluid or the entire organism, constitute the metabolome (Lankadurai et al., 2013), and are likely to contribute to the functional state of cells and serve as a direct signature of biochemical activity (Patti et al., 2012). Considering this, using such an approach for investigating abalones response to hypoxia might result in a better understanding of the biochemical responses and altered metabolites induced by hypoxia.

The aim of this study was to use a multiplatform metabolomics approach, utilising untargeted nuclear magnetic resonance (NMR) spectroscopy, untargeted gas chromatography-time of flight spectrometry (GC-TOF), semi-targeted liquid chromatography-quadrupole time of flight mass spectrometry (LC-QTOF) analyses of butylated esters, semi-targeted gas chromatography-mass spectrometry-detector (GC-MSD) analyses of fatty acid methyl esters (FAMEs) and targeted liquid chromatography-tandem mass spectrometry (LC-MS/MS), to generate a holistic view of the metabolic pathways affected by functional hypoxia, in order to characterise the bioenergetics of abalone metabolism, and to evaluate the interplay of various hypoxic tissues (adductor muscle, foot muscle, epipodial tissue, haemolymph, left gill and right gill) in *H. midae*.

## RESULTS

### Heatmap visualisation of data

**Fig. 1.**
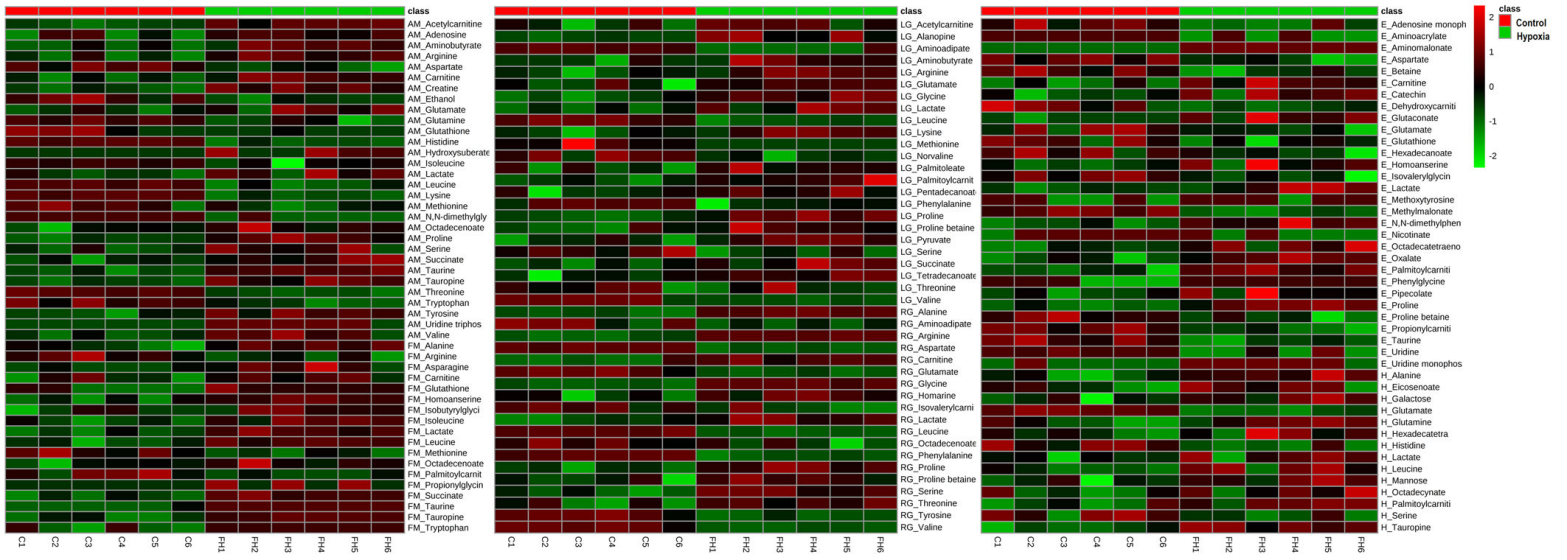
**Heatmap visualisation of metabolomics data generated from *H. midae* following functional hypoxia.** Each row represents a significant (*P*<0.05) metabolite detected in abalone adductor muscle (AM), foot muscle (FM), left gill (LG), right gill (RG), haemolymph (H) and epipodial tissue (E) samples, and each column represents the samples of control (C) and functional hypoxia (FH) groups, respectively.

The metabolites that differed significantly between the control and functional hypoxia group (*P*<0.05 and *d*>0.8) are shown in the heatmap presented in Fig. 1. Each row represents the metabolite detected in adductor muscle (AM), foot muscle (FM), left gill (LG), right gill (RG), haemolymph (H) and epipodial tissue (E) samples, and each column represents the samples of control (C) and functional hypoxia (FH) groups, respectively.

### Tissue-specific metabolite findings of *H. midae* subjected to functional hypoxia

Additionally the significant metabolites are listed in Table 1. Starting with the findings of the adductor muscle sample, information on the metabolite of interest in terms of *P*-value (classified only as *P*<0.05, *P*<0.001 or *P*<0.0001), *d*-value, an increase (↑) or decrease (↓) in relative intensity, the identification level (ID) assigned, the analytical platform on which the metabolite was detected and the metabolite class best describing the finding are shown. Next, information on the metabolites detected in the foot muscle, epipodial tissue, haemolymph, left gill and right gill samples can be found.

**Table 1.**
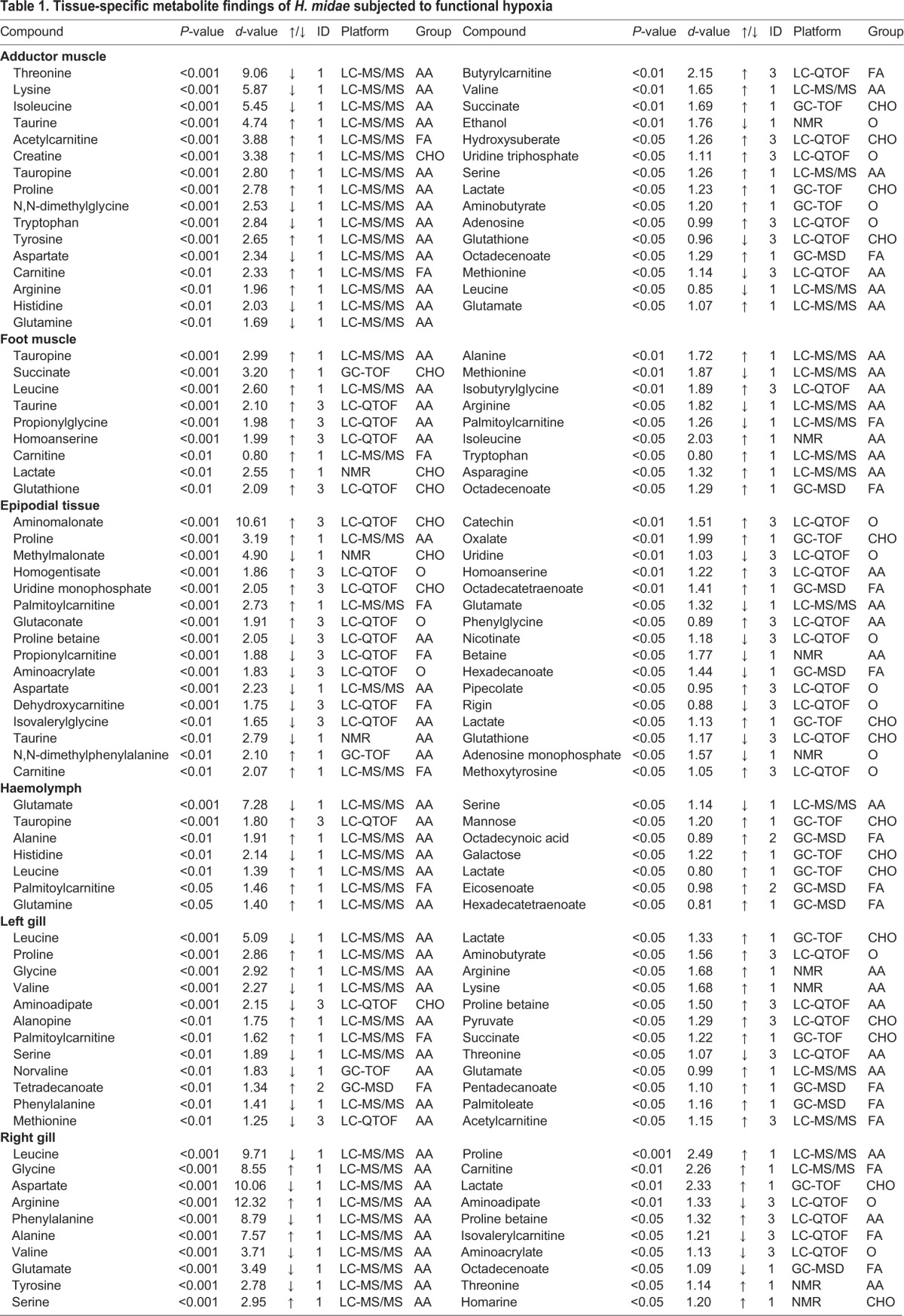
**Tissue-specific metabolite findings of *H. midae* subjected to functional hypoxia**

### Metabolic map of *H. midae* metabolic response following functional hypoxia

**Fig. 2.**
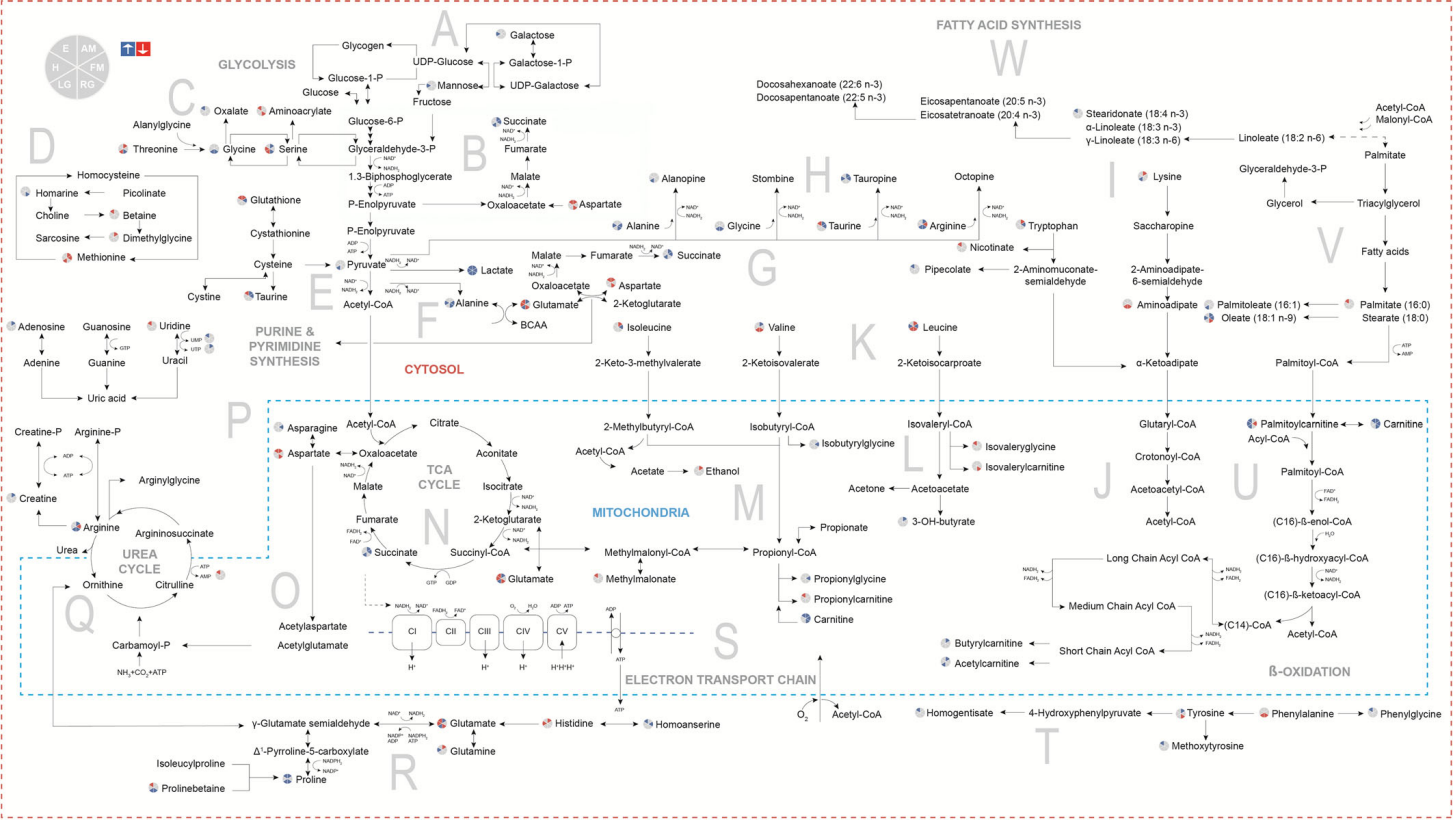
**Metabolic map of *H. midae* metabolic response following functional hypoxia.** Elevated (blue) and decreased (red) metabolite findings in abalone adductor muscle (AM), foot muscle (FM), left gill (LG), right gill (RG), haemolymph (H) and epipodial tissue (E) following functional hypoxia.

Subsequently the data presented in Fig. 1 and Table 1 were mapped on a metabolic chart as depicted in Fig. 2. Metabolites affected by hypoxia are indicated as elevated (blue) or decreased (red) metabolite abundance when directly compared to the findings of the control group. Findings are represented by the tissue [adductor muscle (AM), foot muscle (FM), left gill (LG), right gill (RG), haemolymph (H) and epipodial tissue (E)] in which the finding was made when viewing the key next to the metabolite. Manual pathway analysis was done by focussing on those pathways that showed significant change during hypoxia. A pathway was considered important when most of the metabolites in the pathway were affected, and/or when the shared metabolites in the pathway (i.e. metabolites connected with other pathways) were also considerably altered. The pathways affected by functional hypoxia (Fig. 2) included: (A) sugar metabolism (glycolysis); (B) oxaloacetate-succinate pathway; (C) threonine-serine-glycine metabolism; (D) sulphur-containing amino acid metabolism; (E) cysteine-taurine-cysteine metabolism; (F) pyruvate-lactate/alanine pathway; (G) aspartate-succinate pathway; (H) pyruvate-opine pathway; (I) lysine-tryptophan metabolism; (J) glutaryl-CoA pathway; (K) branched chain amino acid metabolism; (L) ketone body metabolism; (M) propionyl-CoA pathway; (N) tricarboxylic acid (TCA) cycle; (O) alternative aspartate pathway; (P) purine and pyrimidine metabolism; (Q) urea cycle; (R) proline metabolism; (S) electron transport chain (ETC); (T) phenylalanine-tyrosine metabolism; (U) beta(β)-oxidation; (V) fatty acid transport pathway; and (W) fatty acid synthesis.

## DISCUSSION

An average of 2036 features was reliably detected for each tissue (after data clean-up). From this, the metabolites that differed markedly between the groups were identified (Table 1) and visualised (Fig. 1). These findings were assessed in a holistic manner by using a metabolic map (Fig. 2) uniquely designed for abalone metabolism, highlighting the anaerobic energy producing systems and the contribution of various macromolecules (carbohydrates, proteins and lipids) as energy substrates for survival during hypoxia.

### Substrate-level phosphorylation for ATP production during functional hypoxia

It is already well known that substrate-level phosphorylation serves as an alternative route for ATP production when mitochondrial respiratory activity becomes inadequate (Feala et al., 2009). Based on the findings of Table 1 (Fig. 1), this study confirms that *H. midae* utilise substrate-level phosphorylation in terms of phosphagen breakdown (Pathway Q) to ensure that energy is instantaneously available (Grieshaber et al., 1993) during the initial phase of functional hypoxia. The presence of creatine in the adductor muscle of the hypoxic abalone suggests that energy is maintained with phosphocreatine metabolism in addition of phosphoarginine, which is more commonly used in marine invertebrates. Based on Fig. 2 (Pathway Q), the contribution of phosphoarginine and phosphocreatine for the production of arginine and creatine during ATP replenishment in the adductor muscle is strongly suggested, considering the elevated levels of these metabolites detected during functional hypoxia.

When oxygen availability is compromised to the level where aerobic ATP production cannot meet the increased demand (fast enough), anaerobic glycolysis becomes the main way to rapidly produce ATP (Garrett and Grisham, 2010), as demonstrated by the elevated levels of lactate, several opines, succinate and alanine detected in selected tissues of *H. midae* (Table 1). Altogether Pathways B, F and H allowed for the regeneration of cytoplasmic nicotinamide adenine dinucleotide (NAD^+^) for usage in the glyceraldehyde-3-phosphate dehydrogenase reaction of glycolysis (Fig. 2). Consequently NAD^+^ feeds into the glycolytic pathway, and enables phosphoglycerate kinase and pyruvate kinase to synthesis ATP directly from the phosphorylation of adenosine diphosphate (ADP) (Garrett and Grisham, 2010). Abundant amounts of pyruvate is produced which is converted to lactate (and opines) while co-enzymes are released to allow glycolysis to continue (Whitney and Rolfes, 2008). This production of predominantly lactate during functional hypoxia is clearly demonstrated in Fig. 2, where lactate was found elevated in all of the tissues investigated (Pathway F). The most notable differences in lactate levels were found in the foot muscle, followed by the right gill, left gill, adductor muscle, epipodial tissue and lastly the haemolymph samples. Another class of end-products of anaerobic glycolysis, called opines, also contributed to the replenishment of NAD^+^ levels during functional hypoxia. Tauropine was predominantly produced by *H. midae* following functional hypoxia (Pathway H), detected in highest levels in the foot muscle, adductor muscle and then haemolymph samples. Alanopine was also detected as elevated in the left gill sample (Table 1). Interestingly, no octopine (formed from the condensation of pyruvate and arginine) was detected in *H. midae* exposed to functional hypoxia (Pathway H), despite arginine availability in these tissues. This is most likely due to the low enzyme activity of octopine dehydrogenase in this species (O'omolo et al., 2003). These results might also indicate that functional hypoxia (Fig. 2) is not severe enough for *H. midae* to require octopine synthesis. It has previously been reported, that octopine formation only occurs during recovery from functional anaerobiosis in most *Pecten* species (Gäde et al., 1984). Following physical activity, octopine dehydrogenase was however detected in *H. kamtschatkana* (Donovan et al., 1999) and *H. midae* (O'omolo et al., 2003) without allowing some time for recovery. Contradictory to previous research reporting elevated concentrations of lactate and tauropine in both the adductor and foot muscles in the abalone *H. lamellosa* (Gäde, 1988), *H. discus hannai* (Sato et al., 1991), *H. iris* (Baldwin et al., 1992) and *H. midae* (O'omolo et al., 2003) after functional hypoxia, we indicate the highest levels of lactate and tauropine in the foot muscle samples analysed (Fig. 2). Previous reports speculate that the shell adductor muscle is the main source of tauropine production during exercise, as it enables the animal to re-attach itself after becoming dislodged. In *H. kamtschatkana*, in fact, a fivefold increase in the adductor muscle tauropine was reported, leading to the argument that anaerobic glycolysis supplies some of the much needed energy required in the adductor muscle, in contrast to that of the foot muscle, which primarily produces energy aerobically (Donovan et al., 1999). Seeing that lactate was elevated in all of the tissues investigated in our study, while tauropine was only elevated in half of the investigated tissues, following functional hypoxia (Table 1), it suggests a gradual and progressive requirement to correct the redox imbalance during prolonged exposure to hypoxia. As functional hypoxia persists, replenishment of NAD^+^ via lactate alone becomes insufficient, leading to the synthesis of opines. Also the release of NAD^+^ via the production of succinate (Pathway B) assists with substrate availability for ATP production during functional hypoxia. Increased adductor muscle and foot muscle succinate in *H. midae* corresponds to findings in *H. lamellose* where a synchronised depletion of aspartate accompanied by elevations in alanine and succinate in the foot and adductor muscles were reported in response to anoxia (Gäde, 1988).

### Response of protein metabolism to functional hypoxia

Of all the detected compounds classes, proteins (amino acid subgroup) are the group of metabolites most changed by functional hypoxia (Table 1). Considering Fig. 2, it becomes apparent that proteins are used for energy production during functional hypoxia. Abalone [marine invertebrates in general (Fields, 1983)] tissue are noted for high concentrations of amino acids, thus the use of protein for energy metabolism directly foreseeing the conversion to other amino acids or to glucose (Shao et al., 2015) is possible in this functional hypoxic scenario.

Several glucogenic amino acids (alanine, arginine, glycine, proline and serine) were elevated in the right gill tissue samples analysed from the functional hypoxic group (Fig. 2, Table 1). Amino acids, threonine, glycine and serine, feed into the glyceraldehyde-3-phosphate reaction of the glycolysis pathway (pathway E), adding to energy production in especially the right gill, which was severely affected by functional hypoxia (Fig. 1). Typically, during stressed conditions (like functional hypoxia) glucogenic amino acids are converted to alanine via the transamination of pyruvate (Pathway F) (Salway, 2004). Increased alanine was observed in the right gill, foot muscle and haemolymph samples, largely due to the fact that alanine is in equilibrium with pyruvate, which is oxidatively decarboxylated to carbon dioxide (CO_2_) and acetyl coenzyme A (CoA) in aerobic conditions (Salway, 2004). However, in anaerobic conditions, abalone can also use the increased alanine for alanopine production.

Functional hypoxia resulted in elevated asparagine in the foot muscle and reduced levels of aspartate in epipodial tissue, adductor muscle and the right gill samples analysed (Pathway O), supporting the fact that anaerobic catabolism of aspartate is commonly implemented by marine invertebrates. Aspartate in particular is catabolised by reactions of the TCA cycle running from oxaloacetate to succinate (Storey, 2005). Thus, the decrease in aspartate can be ascribed to the replenishment of the TCA intermediate, oxaloacetate. Alternatively, it can also be a result of the apparent unchanged TCA cycle, which does not lead to accumulation of connecting compounds. Aspartate is also used for the synthesis of glutamate via the aspartate aminotransferase reaction, and serves as the precursor for the biosynthesis of a number of other amino acids (Shao et al., 2015). The amino acid glutamate (Pathway G and R) was increased in the left gill and adductor muscle samples, but decreased in the right gill, haemolymph and epipodial tissue samples. Glutamate not only plays a vital role as amino donor via aminotransferase reactions enabling amino acid biosynthesis (Venter et al., 2016b), but also serves as a precursor for glutamine synthesis, and is overall involved in cellular metabolism (Shao et al., 2015). Glutamine (Pathway R) was increased in the haemolymph sample and decreased in the adductor muscle tissue (the exact opposite of glutamate) demonstrating interplay with glutamate.

The branched-chain amino acids (BCAA) leucine and isoleucine were reduced in the adductor muscle samples (Pathway K), while valine was elevated, following functional hypoxia (Fig. 2). During periods of extended exercise (functional hypoxia), BCAAs are used as carbon skeletons in gluconeogenesis and alanine synthesis in order to prevent lactate accumulation (Layman, 2002). Conversely, elevated BCCAs were also reported in the gills of *H. diversicolor* (Lu et al., 2016) and *H. fugens* (Tripp-Valdez et al., 2017) following hypoxia and thermal stress.

Some amino acids, like glycine and taurine, feed into the glycolysis pathway, eventually assisting with glycolytic ATP production, and are termed osmolytes. Generally, the osmolytes of marine invertebrates are present at higher concentrations due to their importance in the regulation of osmosis (Zhou et al., 2015). However, the osmolytes betaine and dimethylglycine (Pathway D) were lower as a result of functional hypoxia, which might have drained these compounds in an attempt to regulate the osmotic pressure. Then again, increases in alanine, taurine, alanopine, tauropine and other non-essential amino acids detected in this study complies with maintenance of intracellular osmotic pressure in abalone (Rosenblum et al., 2005; Tripp-Valdez et al., 2017). An increased presence of the osmolyte homarine (an endogenously synthesised hetero-aromatic quaternary ammonium compound) was detected in the right gill of abalone subjected to functional hypoxia (Pathway D), which is most likely synthesised via methylation of picolinate or via the metabolism of glycine and succinyl-CoA (Netherton and Gurin, 1982). The exact role of hormarine in abalone metabolism is not yet known despite it being elevated in *H. iris* muscle tissue after exposure to wave action (Wells et al., 1998), the foot muscle tissue and digestive glands of stunted and diseased *H. rufescens* (Viant et al., 2003), and digestive gland and foot muscle tissue of Rickettsiales-like prokaryote-infected *H. rufescens* (Rosenblum et al., 2005). Homarine has previously been implicated to assist with osmoregulation and trans-methylation, and exhibits morphogenetic activity which may influence cell proliferation and differentiation (Rosenblum et al., 2005). The elevated levels of homarine detected in the right gill of *H. midae* after exposure to functional hypoxia in this experiment (Fig. 2) support its osmoregulatory function, since this tissue will be the first to be influenced by oxygen deprivation. Proline-betaine, another osmolyte (Hayat et al., 2012), was detected in elevated levels in the gills of the abalone after functional hypoxia (Table 1), and can in effect be used to produce proline (Pathway R) also elevated in the gill samples. Furthermore, proline resulted in significant increases in the adductor muscle and epipodial tissue samples of *H. midae*. Typically, proline acts as an osmolyte to balance water stress and possesses protective mechanisms for the stabilising of proteins and antioxidant enzymes. The scavenging of reactive oxygen species and balancing of redox homeostasis are also promoted by proline metabolism (Liang et al., 2013).

### Response of carbohydrate and lipid metabolism to functional hypoxia

Carbohydrates are deemed as the most important energy source for abalone and therefore are supplied in bulk to abalone formulated feeds (Lee, 2004). When ATP is required quickly, during episodes of intense exercise, carbohydrates are primarily utilised as they support maximal rates of ATP production (Weber, 2011). During physical activities, glycogen (the stored form of glucose) is broken down to supply glucose (Whitney and Rolfes, 2008). Based on the findings, the glycolysis pathway in Fig. 2 and Table 1 suggests that carbohydrates were metabolised for anaerobic energy production. When evaluating the presence of substrates for glycolytic use, only galactose and mannose (Pathway A) were elevated in the haemolymph samples. Also the intermediates of the TCA cycle (with succinate being the exception) did not vary significantly between the experimental groups, suggesting that the TCA cycle functioned adequately despite a redox imbalance (skewed NAD^+^:NADH ratio). However, the presence of lactate, alanopine and tauropine indicates the use of anaerobic rather than aerobic carbohydrate metabolism following functional hypoxia. Furthermore, the accumulation of these anaerobic end products demonstrate that the rate of pyruvate production exceeded the rate of pyruvate clearance, which creates a view that functional hypoxia resulted in a rapid decrease in cellular ATP, which was compensated for by anaerobic ATP production.

The most apparent changes in the fatty acid profile of *H. midae* after the functional hypoxia experiment (Table 1), were the elevated levels of palmitoleate (C16:1) in the left gill, and oleate (C18:1 n-9) in the muscle and haemolymph samples (Pathway V). In *H. discus hannai* and *H. diversicolor*, oleate was also reported to be the predominant fatty acid released from lipids for β-oxidation (Lou et al., 2013; Li et al., 2015)*.* A few fatty acids and carnitines were affected by functional hypoxia (Fig. 2), corresponding to the relatively low lipid stores previously reported to occur in abalone tissue (Vosloo and Vosloo, 2010). An increase in palmitoleate supports the increases observed in palmitoylcarnitine, carnitine and acetlycarnitine, which demonstrates compromised β-oxidation (Pathway U), despite fatty acid release from lipids through activated lipase action.

### Abalone tissue-specific responses and interplay

A general observation was that the largest number of significantly changed metabolites was detected in the adductor muscle, followed by the gills, the foot muscle, epipodial tissue and lastly the haemolymph, which displayed the least number of significant metabolites between experimental groups (Fig. 1, Fig. 2 and Table 1). The results indicated that *H. midae* tissues display a diverse response to hypoxia, most likely attributed to the varying functionality and metabolism associated with the various organs from which the tissue was sampled. Additionally, much like higher organisms, certain metabolites are shuttled between various different organs and tissues (e.g. from hypoxic tissue to regions that have access to oxygen) where they can be more appropriately utilised. An example of this in higher animals is the lactate shuttle between fatigued muscles and the liver. Each tissue function, in terms of the hypoxia, will subsequently briefly be discussed.

#### Gills

Seeing that environmental oxygen levels did not change, the fact that the gills displayed alterations following functional hypoxia are likely attributed to the shuttle of accumulated products from systemic circulation. Hence, it can be reasoned that aerobic metabolism in the gills still function adequately. Because of this, the gills might be a site where accumulated products in the circulation are further catabolised (like NADH), which is shuttled through increased perfusion. The gills can be regarded as the first line of physiological mechanism that enable abalone to cope with variation in oxygen levels (Ragg and Taylor, 2006; Morash and Alter, 2016). The right gill, which is generally perfused, serves as sole handler to the initial change in internal oxygen levels, explaining why large metabolic changes were detected in the right gill. After a period of increased oxygen demand, more haemolymph can be diverted to the left gill for increased oxygen uptake (Ragg and Taylor, 2006). The metabolite arginine, detected in the right gill, displayed the largest increase in metabolic activity (Table 1), possibly due rapid energy production via phosphoarginine. It is also likely a result of shuttled arginine as the gills can either release or use this product. Then again, aspartate, measured in the right gill, showed the largest decrease in metabolic response. Considering the involvement of aspartate in the synthesis of TCA cycle intermediates, lower levels are expected in scenarios where oxygen supply is still adequate for aerobic metabolism. In the abalone *H. diversicolor* the gills were also reported as easily affected following hypoxic stress (Lu et al., 2016).

#### Haemolymph

During intense activity, the abalone circulatory system is known to favour oxygen storage instead of oxygen delivery resulting in insufficient haemolymph gas transport and in effect isolation of general circulation (Donovan et al., 1999). Of all the samples studied, the least number of metabolites were detected in the haemolymph (Table 1) and of the detected metabolites, most were elevated (Fig. 2). During functional hypoxia, elevated energy demand results in the elevated release of metabolites from cellular stores that are shuttled between various organs. Metabolic end products produced during functional hypoxia may be released into the haemolymph and absorbed by other tissues (organs), which are able to oxidise these products (metabolic shuttle). Haemolymph also represents the exo-metabolome since it contains metabolite information of how the intracellular metabolic network influences its external environment (Dunn, 2008). Furthermore, the haemolymph of abalone utilise a reverse Bohr shift mechanism which utilises the decrease in pH during anaerobic conditions to ensure survival for longer in the absence of oxygen (Venter et al., 2016b).

#### Muscle

Energy production via both substrate-level phosphorylation and the glycolysis pathway (Venter et al., 2016b) require metabolic fuel, and as abalone muscle has the ability to store large amounts of amino acids and phosphoarginine (more than the other tissues), carbohydrates and proteins are rapidly broken down to serve as substrates for energy production. The abundance of affected muscle metabolites is an expected outcome following functional hypoxia, considering the reduced oxygen supply to the muscle, forcing the muscle to switch to anaerobic metabolism as activity endures. The findings of both adductor and foot muscle predominantly displayed changes in metabolites detected in the functional hypoxia group most likely due to inhibition of mitochondrial metabolism and fatigue following functional hypoxia. This demonstrates the reliance of abalone muscle on oxygen for energy metabolism, despite its capacity for anaerobic metabolism, as confirmed in previous experiments on *H. lamellose* (Gäde, 1988).

#### Epipodial tissue

Unique findings made by observations in the epipodial tissue include betaine, uridine, adenosine monophosphate, oxalate and nicotinate, which amongst other functions support pyrimidine nucleotide synthesis and participate in redox reactions (Young et al., 2017). However, most of the metabolite results of the epipodial tissue, such as aspartate and carnitine, are similar to those in other tissues, suggesting that the epipodial tissue has similar metabolic profiles to the adductor muscle and right gill. This presents an important opportunity for non-destructive sampling for health screening, without critical damage to the animal (Wasko et al., 2003; Slabbert and Roodt-Wilding, 2006). Also, the role of epipodial tissue as a means to shuttle metabolites cannot be dismissed based on the findings made here.

*H. midae* tissue display a diverse response to functional hypoxia, most likely attributed to the varying functionality and metabolism associated with the various organs from which the tissue was sampled. Additionally, much like higher organisms, certain metabolites are shuttled between various different organs and tissues (e.g. from hypoxic tissue to regions that have access to oxygen) where they can be more appropriately utilised (Brooks, 1986; Proia et al., 2016).

## CONCLUSIONS

Taken together, this study brings to front the metabolic response of *H. midae* after subjection to functional hypoxia, demonstrating how abalone can cope with episodes of burst activity (functional hypoxia), by continuing to drive the metabolic needs via anaerobic metabolism. Energy is initially produced by means of phosphoarginine breakdown, followed by anaerobic central carbon metabolism, with the production of lactate, opines (tauropine and alanopine) and succinate. It is also clear that *H. midae* used all of the main metabolite classes, e.g. proteins (amino acids), carbohydrates (glucose) and lipids (fatty acids), to contribute to ATP production when exercise is induced. By performing cross-tissue analysis it becomes evident that all of the investigated tissues are not affected to the same extent following functional hypoxia. The gills and haemolymph manage oxygen delivery based on oxygen demand. The haemolymph also shuttles metabolites between tissues where some metabolites are further metabolised. Both adductor and foot muscle produced anaerobic end-products releasing NAD^+^ for glycolytic ATP production, making it possible to say that *H. midae* is more dependent on anaerobic metabolism after a period of induced functional hypoxia. No special metabolic function apart from metabolite shuttling was observed in the epipodial tissue, but it does correlate well with other tissues making this an important tissue for non-destructive research purposes.

Functional hypoxia can be seen as a self-inflicted stressor, but *H. midae* has shown to have well-developed metabolic capabilities to withstand the exercise-induced episode, whereafter normal aerobic metabolism will likely continue once oxygen availability returns. Ideally in an aquatic system, stress should be limited to ensure that metabolic energetics are used for anabolic processes resulting in growing organisms, and not catabolic processes aimed at energy production for survival, like typically experienced in this metabolomics study. The use of metabolomics enabled a global view of the metabolic response implemented by *H. midae* adductor muscle, foot muscle, left gill, right gill, haemolymph and epipodial tissue samples, following exposure to functional hypoxia. With this in mind, it is important to next focus on certain metabolic intermediates and/or end-products (e.g. arginine, lactate, opines) and establish defined concentration ranges for normality and certain stressors. This may serve well to monitor and assess abalone health on abalone farms, which is still lacking in the aquaculture industry (Fasulo et al., 2012; Alfaro and Young, 2016).

## MATERIALS AND METHODS

### Experimental setup and animals

Adult abalone were collected during the winter season from the grow-out platform at HIK Abalone Farm (Pty) Ltd, situated in Hermanus, South Africa. As part of day-to-day farming activities, the tanks were cleaned weekly and animals were provided with standard artificial abalone feed (Vosloo et al., 2013). All of the research pertaining to the perturbations and abalone collection was done in accordance with institutional guidelines of the relevant institutional committees and granted Aquaculture Research Permit. Animals (*n*=12) were removed from the same raceway and placed into a large aquarium tank with flowing seawater. The control animals (*n*=6) were removed and dissected for sampling of adductor muscle, foot muscle, left gill, right gill, epipodial tissue and haemolymph samples. In order to induce functional hypoxia, the remaining animals were placed upside-down on their shells (O'omolo et al., 2003). After relatively vigorous contractions of the shell adductor muscle, the animals eventually regained their normal posture, and were immediately inverted again; this was repeated to the point where the animals were unable to upright themselves. After 18 min the abalone were considered exhausted and were removed from the system and dissected in the same manner as the control group. Prior to dissection, the animals were weighed to the nearest 0.01 g and the shell lengths were measured to the nearest 0.10 mm, along the longest axis, using callipers. Additionally, abalone gender was assessed by observing the gonad colour, which is green in females and cream in males. The control group had an average wet weight of 83.00±8.27 g and shell length of 75.53±2.05 mm, and consisted of four males and two females. The functional hypoxia group had an average wet weight of 94.33±11.13 g and shell length of 79.00±2.76 mm and consisted of two males and four females.

### Sample dissection

All abalone were collected, dissected and snap frozen within a standardised time period (Hernandes et al., 2017), accumulating to approximately 10 min per animal. Abalone were shucked individually with a scalpel, working rapidly from the anterior to posterior axis, cutting longitudinally through the foot between the mantle and the distal surface of the foot. Haemolymph was collected from the pedal sinus using a 1 ml syringe with a 27-gauge needle and immediately transferred and stored in micro-centrifuge tubes. Epipodial tissue was cut from the left anterior of the area surrounding the abalone foot muscle using dissection scissors. Both the left and the right gill were collected, by making an incision in the mantle situated to the left side of the animal, located directly under the shell pores. The gills were carefully removed with dissection forceps, from the left and right walls of the mantle cavity. A sample of the shell adductor muscle was removed using a scalpel, from the ventral surface towards the central point where the muscle attaches to the shell. The foot muscle samples were collected from the posterior side of the ventral surface, excluding sole epithelium and majority of the mucus glands. Muscle samples to the equivalent of the volume of a micro-centrifuge tube were sampled in small blocks using a scalpel. As soon as the sample of interest was removed, it was immediately placed into a micro-centrifuge tube and snap frozen using dry ice.

### Metabolite extraction

Tissue homogenisation was performed using the two-step method described by Venter et al. (2016a), using a sample weight of 20 mg for the right gill tissue, 40 mg for the left gill tissue, 30 mg for the epipodial tissue and 60 mg for the adductor and foot muscle tissue.

For every 10 mg of abalone tissue, 20 µl water, 50 µl methanol and 20 µl internal standard (2-acetamidophenol and 3-phenylbutyric acid, with a final concentration of 50 µg/ml) was added along with both a 3- and a 7-mm stainless steel bead to micro-centrifuge tubes. Samples were homogenised for 4 min at 30 Hz using a Retch M400 vibration mill, whereafter 20 µl chloroform and 10 µl water were added to the homogenate. The samples were vortexed for 1 min and incubated on ice for 10 min, before they were centrifuged at 25,000×***g*** for 10 min at 4°C to induce phase separation. Selected volumes of the aqueous and organic phases were transferred to a screw-top vial (depending on the analytical platform) and dried under a gentle stream of nitrogen at 37°C, together with an additional 100 µl platform-specific internal standard. For NMR analysis, 100 µl of the polar phase and 50 µl of the a-polar phase were transferred, and dried together with nor-leucine internal standard (100 µg/ml). For LC-QTOF analysis, 70 µl of the polar phase and 35 µl of the a-polar phase were transferred, and dried together with nor-leucine internal standard (100 µg/ml). For GC-QTOF analysis, 70 µl of the polar phase and 35 µl of the a-polar phase were transferred, and dried together with nonadecanoic acid internal standard (100 µg/ml). For LC-MS/MS analysis, 10 µl of the polar phase and 5 µl of the a-polar phase were transferred, and dried together with nor-leucine internal standard (10 µg/ml). For GC-MSD analysis, nonadecanoic acid internal standard (100 µg/mL) and 150 µl of the a-polar phase of the tissue homogenate were dried. All samples were dried under a gentle stream of nitrogen at 37°C.

Haemolymph samples were prepared by adding 500 µl of the collected haemolymph to 1500 µl cold acetonitrile in micro-centrifuge tubes. The samples were vortexed and incubated on ice for 30 min, followed by centrifugation at 25,000×***g*** for 10 min at 4°C. The supernatant was aliquoted into various screw-top vials (one for each analytical platform). After the addition of 100 µl instrument specific internal standard, all samples were dried under a gentle stream of nitrogen at 37°C.

### Sample preparation

Dried sample extracts for NMR spectroscopy analyses were re-dissolved in 500 µl ultra-pure water and centrifuged at 12,000×***g*** for 10 min, whereafter the ultra-filtrate was transferred together with 50 µl 1.5 M potassium phosphate monobasic deuterated NMR buffer solution at pH 7.4 (Dona et al., 2014) to 5 mm NMR glass tubes.

The sample extracts prepared for untargeted GC-TOF analyses were removed from the freezer and dried under a nitrogen stream at 60°C for 5 min, prior to oximation and silylation. A volume of 50 μl of the oximation reagent (200 mg methoxyamine dissolved in 10 ml pyridine) was added to the samples, whereafter the samples were capped, vortexed for 1 min and incubated for 60 min at 60°C. Once oximation was completed, the samples were cooled prior to adding 100 μl O-bis(trimethylsilyl)trifluoro acetamide (BSTFA), containing 1% trimethylchlorosilane (TMCS). The samples were then vortexed for 1 min and incubated for 60 min at 60°C and transferred to glass GC vials for analyses (Venter et al., 2016a).

Samples prepared for LC-MS/MS and LC-QTOF analyses were butylated once the dried residue (stored at −80°C) was dried again, using nitrogen at 60°C for 5 min. To the dried residue, 100 µl 3 N butanolic hydrogen chloride (HCl) was added and the samples were incubated at 60°C for 60 min. Butanolic HCl (3 N) was prepared by creating a 1:4 ratio of acetyl chloride and 1-butanol solution. The butylated samples were then evaporated to dryness again under a stream of nitrogen at 60°C. The dried residue was reconstituted in 200 μl water:acetonitrile (50:50) containing 0.1% formic acid (Venter et al., 2017).

The GC-MSD-analyses-prepared dried extracts were removed from the freezer and dried again using a stream of nitrogen at 60°C for 5 min. The samples were suspended in 500 µl chloroform and 500 µl methanol, followed by the addition of 1 ml of methanolic potassium hydroxide (KOH), and incubated for 30 min at 40°C. Methanolic KOH was prepared daily before analysis of the batch, by dissolving 280 mg of potassium hydroxide pellets in 25 ml methanol (Lindeque et al., 2013). Once the samples were cooled to room temperature, FAMEs were extracted with the addition of 2 ml hexane, 200 µl acetic acid (1 N) and 2 ml water. The samples were centrifuged for 5 min at 2000×***g*** in order to separate the organic and water phases. The upper organic phase was recovered using a clean tube with a rubber suction teat, and the lower phase was re-extracted with 2 ml hexane, for another three cycles as described above. The pooled organic solvent phases collected were evaporated under a gentle stream of nitrogen at 37°C. Following the drying step, 50 μl oximation reagent (200 mg methoxyamine dissolved in 10 ml pyridine) was added, then vortexed for 1 min and incubated for 60 min at 60°C. After cooling, silylation was performed by adding 50 μl BSTFA (containing 1% TMCS) to the samples and incubated for 60 min at 60°C and transferred to glass GC vials for analyses (Willers et al., 2016).

### Sample analysis

Samples collected from the same tissue were assigned to the same batch using a randomisation equation to allocate these to the respective batches for analysis (Dunn et al., 2012), with quality control samples included amongst the batch which was injected at regular intervals throughout the analytical run of the analysed batch. Thus, six batches (adductor muscle, foot muscle, left gill, right gill, haemolymph and epipodial tissue) were analysed over 6 days on all of the analytical platforms described below.

NMR spectroscopy analyses were performed on a Bruker Advance III HD NMR spectrometer operating at 500 MHz ^1^H, equipped with a triple-resonance inverse (TXI) ^1^H(^15^N, ^13^C) probe head and x, y, z gradient coils. Automated tuning and matching, locking and shimming using the standard Bruker routines ATMA, LOCK, and TopShim were used to optimise the NMR conditions daily. The NMR spectra were acquired as 128 transients in 32,000 data points with a spectral width of 6002 Hz. The sample temperature was maintained at 27°C and the water resonance was pre-saturated by single-frequency irradiation during a relaxation delay of 4 s, with a 90° excitation pulse of 8 μs, acquiring spectra at 64 scans per sample (∼runtime of 8 min per sample). Shimming of the sample was performed automatically on the deuterium signal. The resonance line widths for trimethylsilyl-tetradeuteropropionic acid and metabolites were <1 Hz (measurements at half the height of the peak). Fourier transformation and phase and baseline correction were done automatically. Bruker Topspin (Version 3.1) software was used for spectral data processing and Bruker AMIX (Version 3.9.12) software was used to distinguish and identify metabolites (Ellinger et al., 2013; Irwin et al., 2016).

GC-TOF analyses were executed on a LECO Pegasus HT mass analyser coupled to an Agilent 7890A GC. Chromatographic separation was performed on a Restek RXi-1MS column (30 m×0.32 mm×0.25 μm). A sample volume of 1 μl was injected (using a split/split-less injector) per run with a 1:20 split ratio. The front inlet temperature was kept at 250°C. An initial oven temperature of 80°C was maintained for 1 min and then increased as follows: 10°C/min to 150°C at 1.50 min, 14°C/min to 230°C, 18°C/min to 300°C where it was maintained for 2 min equating to a total run time of ∼20 min per sample. Hydrogen was used as carrier gas at a constant flow of 3.2 ml/min. The transfer line temperature was maintained at 225°C and the ion source temperature at 200°C for the entire run. Acquisition was delayed for the first 1.9 min which served as a solvent delay. Data were captured with an acquisition rate of 20 spectra (40–950 m/z) per second, with a detector voltage of 50 V over the daily tune voltage and electron energy of -70 V.

LC-MS/MS analyses were performed in positive ion mode using an Agilent 1200 LC system coupled to an Agilent 6410 Triple Quadrupole. A sample injection volume of 0.5 µl was separated chromatographically on an Agilent SB-Aq column (2.1×100 mm×1.8 µm) at 45°C. The mobile phases consisted of (A) water and (B) acetonitrile both with 0.1% formic acid. The gradient separation conditions included: 0 min 5% (B); 0-0.2 min 5% (B); 0.2-2 min 25% (B); 2-7 min 25% (B); 7-7.5 min 90% (B); 7.5-9 min 90% (B) all using a flowrate of 0.4 ml/min; 9-9.1 min 90% (B); 9.1-12 min 95% (B) and 12-12.5 min 5% (B) using a flowrate of 0.5 ml/min, with a post-run step of 4 min at this condition, resulting in a run time of 16.5 min per sample. The drying gas temperature was set at 300°C with a drying gas flow of 7.5 l/min and nebuliser pressure of 30 psi. Selected metabolites (Table S1) were analysed in multiple reaction monitoring (MRM) mode with a dwell time of 45 ms and enhanced sensitivity at an electron multiplier voltage of 400 above tune voltage.

LC-QTOF analyses were conducted using an Agilent 1200 LC system using an injection volume of 5 μl for chromatographic separation. Butylated samples were separated on an Agilent ZORBAX SB-Aq C18 column (2.1×150 mm×3.5 μm) fitted with a guard pre-column at 30°C. The mobile phases consisted of (A) water and (B) acetonitrile, both containing 0.1% formic acid. The separation was performed using the following gradient: 0-3 min 0% (B), 3-11 min 25% (B), 11-30 min 100% (B) at a flowrate of 0.25 ml/min, whereafter the flowrate was increased to 0.5 ml/min to 32 min 100% (B), and ending at 33 min 100% (B) 0.25 ml/min along with a 10 min post-run at this condition, resulting in a runtime of 43 min per sample. Mass spectrometry detection was performed on an Agilent 6510 QTOF mass analyser using positive electrospray ionisation with a drying gas temperature of 300°C, a drying gas flow of 7.5 l/min and nebuliser pressure of 30 psi. The Q-TOF was set to scan from 50 to 1 000 m/z. Both centroid and profile data were stored and the instrument set to extended dynamic range (2 GHz). The all ions MS/MS functionality was used with the following collision energies: 0, 10, 20 and 40 V using nitrogen collision gas. A reference solution containing masses 121.050873 [M+H]^+^ and 922.009798 [M+H]^+^ were constantly infused as accurate mass reference. Furthermore the instrument was calibrated with Agilent ESI-L low concentration tuning mix before the analysis of each batch of samples (Venter et al., 2017).

GC-MSD analyses of FAMEs were performed with an Agilent GC-MSD instrument consisting of a gas chromatograph with a split/split-less injector (250°C) equipped with an auto sampler coupled to an inert XL mass selective detector. The gas chromatograph was equipped with a SPB-1 column (60 m×0.25 mm×0.250 μm film thickness) using helium as the carrier gas at a flow rate of 1.0 ml/min. A sample volume of 2 μl was injected per run with a 1:2 split ratio. The front inlet temperature was kept at 250°C throughout the entire run. The initial oven temperature was 150°C for 4 min, ramping to 200°C at 4°C/min to 250°C at 2°C/min, and then to 295°C at 10°C/min followed by a hold for 2 min, resulting in a runtime of 48 min per sample. The transfer line temperature was set to 280°C and the source temperature at 230°C. Acquisition was delayed for the first 5 min serving as a solvent delay. Data were captured with an acquisition rate of 3.58 spectra (40-450 m/z) per second, with electron energy of 70 eV (Willers et al., 2016).

### Data analysis

NMR data were extracted and pre-processed using Bruker's Topspin NMR software (Version 3.1) as previously described (Irwin et al., 2016). GC-TOF data were extracted using ChromaTOF (Version 4.50.8) as previously described (Venter et al., 2016a). LC-MS/MS data were quantified using Agilent's MassHunter Quantitative software (Version B.06). LC-QTOF data extraction was performed using Agilent's MassHunter Qualitative software (Version B.06) utilising the molecular feature extraction algorithm and find by formula function corresponding to theoretical chemical formula of butylated metabolites listed in an in-house butyl ester library. Agilent's MassHunter Mass Profiler Professional (Version B.02.02) was used to align the data. GC-MSD data were extracted as previously described (Willers et al., 2016). The GC-MSD compound list was then used in MET-IDEA (METabolomics Ion-based Data Extraction Algorithm) (Lindeque et al., 2013) to reintegrate target peaks, to yield a data matrix with no missing values.

Data pre-processing in the form of zero filtering was performed (Venter et al., 2015) to ensure a more complete dataset. The NMR data were normalised as a relative concentration by using the internal standard 2-acetamidophenol. GC-TOF data were normalised relative to the internal standard 3-phenylbutyric acid (Luier and Loots, 2016). Mass spectrometry total useful signal (MSTUS) was the preferred normalisation method for the data acquired by LC-QTOF, GC-MSD and LC-MS/MS analysis (Venter et al., 2015). Data pre-treatment steps were completed by using the webserver MetaboAnalyst (3.0) (Xia et al., 2015). Missing values (or zeros) were replaced by half of the minimum positive value in the original data. Next, data were transformed using generalised logarithm (glog) (Lindeque et al., 2015). Principal component analysis (PCA) was used to determine whether a natural grouping in the data exists when considering this from a multivariate perspective (Lindeque et al., 2015). Gender was investigated using this approach in order to confirm that the relevant biological variance in the compared groups was not influenced by this confounder. As a result, the grouping displayed visually on the PCAs did not display any favour towards possible confounding factors within the groups of interest.

Univariate statistical analyses were performed with MetaboAnalyst (3.0) (Xia et al., 2015) and MS Excel. Student's *t*-test was used to find significant differences in metabolite levels between the experimental groups. Features with a *P*-value <0.05 (false discovery rate corrected *P*-value) indicated that the features differed significantly between the groups and were labelled as important and not removed from the data (Lindeque et al., 2013). Effect size was calculated (on the remaining features) to ensure practical significance, by determining the absolute difference between the means of the two groups divided by the maximum standard deviation of the two groups. Features with a *d*-value >0.8 were labelled as important and remained in the data matrix for further analysis (Ellis and Steyn, 2003). Effect size is similar to fold change (i.e. the absolute difference between the group means) but scaled to the highest standard deviation instead of the control group mean. The important features were visualised with a heatmap (using auto-scaled data) to show concentration variance between samples and groups.

Following a comparison to several in-house, commercial and public libraries/databases, compiled by injection and analysis of previously purchased or synthesised standards, practical and statistical significant features were given identities (where possible). Each identification was ranked using the identification confidence levels previously described (Schymanski et al., 2014), whereafter the important metabolites were used for pathway analysis. If metabolites were detected on more than one analytical platform, the platform with the highest identification confidence level received preference, and if a duplicate metabolite still existed the metabolite with the best (smallest) *P*-value was selected. A metabolic map of the metabolic response of *H. midae* to functional hypoxia was compiled manually, using previous knowledge, relevant literature and online servers like Kyoto Encyclopaedia of Genes and Genomes (KEGG) and International Union of Biochemistry and Molecular Biology (IUBMB)-Nicholson, where possible. The metabolites are reported as relative compound intensities based on the tissue in which the findings were made represented by adductor muscle, foot muscle, epipodial tissue, haemolymph, left gill and right gill samples.

## Supplementary Material

Supplementary information

First Person interview
