# Supplementary material for: The cross-tissue metabolic response of abalone (Haliotis midae) to functional hypoxia
Source: Biol Open. 2018 Mar 15;7(3):bio031070. doi: 10.1242/bio.031070 (PMC5898262; doi:10.1242/bio.031070)
Supplement: Supplementary information [file biolopen-7-031070-s1.pdf]

FIRST PERSON

First person – Leonie Venter

First Person is a series of interviews with the first authors of a selection of papers published in Biology Open, helping early-career researchers promote themselves alongside their papers. Leonie Venter is first author on 'The cross-tissue metabolic response of abalone (*Haliotis midae*) to functional hypoxia', published in BiO. Leonie is a PhD student in the lab of Dr Zander Lindeque in the Human Metabolomics Department at North-West University, Potchefstroom Campus, South Africa, investigating metabolic-driven processes, primarily with the use of metabolomics and their application to answer everyday aquaculture questions.

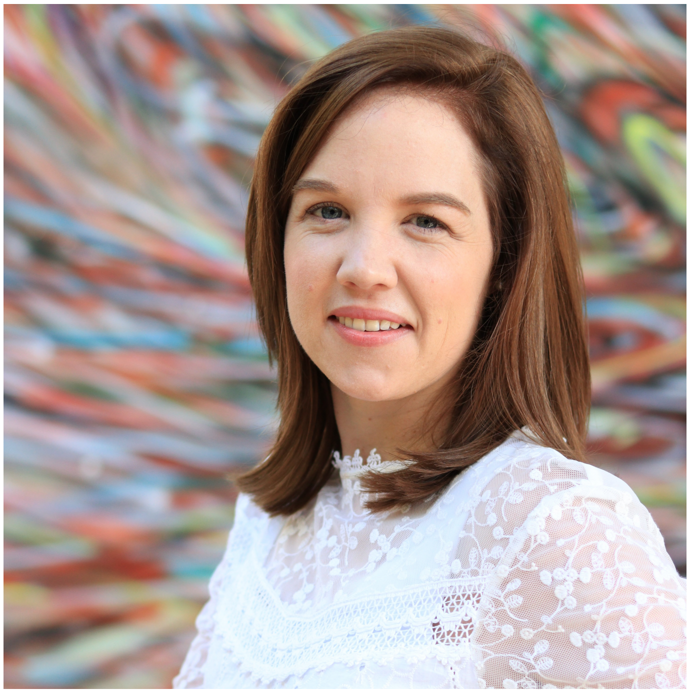

Leonie Venter

What is your scientific background and the general focus of your lab?

My academic background is in biochemistry, with my postgraduate studies focused on the application of metabolomics methods to research questions concerning energy-driven metabolism processes, as is the general focus of the lab. My PhD project involved researching abalone metabolic processes, and expanded my scientific background to aquaculture in the process.

How would you explain the main findings of your paper to non-scientific family and friends?

Metabolism depends on the building and breaking of cellular processes to ensure that energy is created and available for everyday life. Imagine a seesaw on a playground with balanced metabolism in the middle, and building (anabolism) processes on the one side and breaking (catabolism) processes on the other end; whenever the one side of metabolism is high, the other is low. In the context of abalone farming, farmers prefer anabolic reactions to be high, resulting in larger abalone with a higher worth. As soon as a stressor like oxygen shortage (hypoxia) comes into the mix, catabolic reactions start to dominate as other mechanisms now require activation for protection from the stressor. Hypoxia is an episodic occurrence on abalone farms, during which abalone are able to produce energy by using oxygen-independent mechanisms. This is achieved by protein (amino acid), carbohydrate (glucose) and lipid (fatty acid) subunits predominantly stored in the muscle sample of abalone. Additionally, abalone blood proved to function as a shuttle between tissues, transporting metabolites to where they are required for energy production. Metabolic results from abalone gills support oxygen delivery based on demand. Together, tissue interplay enables abalone to manage their energy requirements when hypoxia causes a metabolic imbalance. This is not ideal from a farming perspective but is somewhat reassuring as abalone have well-developed metabolic capabilities to withstand exercise-induced hypoxia, after which balanced metabolism is likely to continue once oxygen availability returns.

Leonie Venter's contact details: North-West University, Potchefstroom Campus, Private Bag X6001, Potchefstroom 2520, South Africa.

E-mail: leonie.venter@nwu.ac.za

What are the potential implications of these results for your field of research?

Now that a metabolic response to functional hypoxia for abalone has been defined, it would make sense to get an idea of what metabolite concentrations would be during standard farming conditions. Following the acquisition of this knowledge, a list of biomarkers needs to be established for use as part of regular health monitoring programmes. Ultimately, the biomarkers of interest should be refined to a point-of-use test that can be used on-farm by general farm personnel. In effect, it would be possible to determine if the metabolism of farmed animals uses the resources supplied for production value, or if these animals are using their metabolic energy to counter stressors, resulting in negative animal growth.

"The compact and clever physical design of abalone came as a total surprise to me. If you think invertebrates can't be that complicated, think again."

What has surprised you the most while conducting your research?

The compact and clever physical design of abalone came as a total surprise to me. If you think invertebrates can't be that complicated, think again. Not only do abalone have well-developed capacities for survival in the absence of oxygen (abalone circulation favours oxygen storage instead of delivery during intense physical activity), but they also implement physiological, cellular and metabolic alterations to

Biology Open (2018): doi:10.1242/bio.031070: Supplementary information

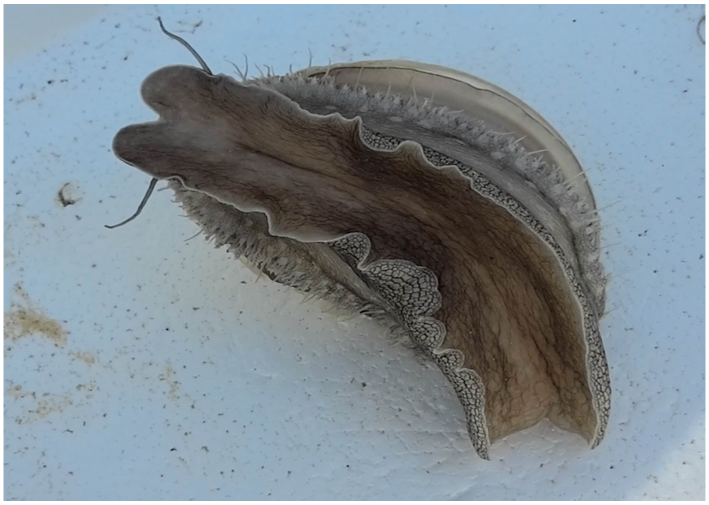

An abalone in the process of regaining normal posture after being placed upside-down on its shell to induce functional hypoxia.

counter hypoxic episodes. The in-depth metabolic changes that they can initiate go beyond what vertebrates are capable of; this is perfectly demonstrated when anaerobic respiration is activated and the production of lactate, multiple opines and succinate ensures energy production. Additionally, their extensive use of amino acids to power multiple metabolic reactions makes you rethink the dietary importance of amino acids in feeds formulated for abalone.

What, in your opinion, are some of the greatest achievements in your field and how has this influenced your research?

The rapidly expanding capabilities and diversity of metabolomics analytical platforms and biostatistics are currently influencing all fields of biology, and their potential in aquaculture is also receiving more attention, placing the research performed in our paper in the spotlight. The fact that compounds can be accurately identified using high-end analytical platforms enabled us to detect opine metabolites in this study, filling some crucial gaps in the abalone metabolic plan. Additionally, the commercial growth achieved by the South African abalone industry is beneficial to research aimed at maximising abalone growth and health. The study serves to address the lack of knowledge of abalone metabolism and determine potential strategies to optimise their metabolism to favour growth.

What changes do you think could improve the professional lives of early-career scientists?

In many countries, there is growing pressure on universities to increase numbers of postgraduate students. This is often detrimental to the mentoring process. Early-career scientists need mentors to invest in their scientific and personal development beyond the project at hand. Only then, the building of your own research prestige becomes more probable, taking pride in doing things well becomes the norm and nurturing of great but realistic research ambitions becomes the goal.

What's next for you?

A postdoctoral position in a new lab as part of a new research team in a new environment is the next step for me. I believe I am now equipped to transfer the skills obtained as part of my PhD to the industry, using metabolomics in association with other omics techniques to assist with biomarker development and/or general health monitoring.

Reference

Venter, L., Loots, D. T., Mienie, L. J., Jansen van Rensburg, P. J., Mason, S., Vosloo, A. and Lindeque, J. Z. (2018). The cross-tissue metabolic response of abalone (*Haliotis midae*) to functional hypoxia. *Biol. Open* **7:** bio031070.
